# Single-cell profiling of microenvironment components by spatial localization in pancreatic ductal adenocarcinoma

**DOI:** 10.7150/thno.73222

**Published:** 2022-06-27

**Authors:** Song Han, Dongtao Fu, Gerik W Tushoski, Lingsong Meng, Kelly M. Herremans, Andrea N. Riner, Thomas J. Geoge, Zhiguang Huo, Steven J. Hughes

**Affiliations:** 1 Department of Surgery, College of Medicine; University of Florida, Gainesville, FL 32610.; 2 Department of Pathology, College of Medicine; University of Florida, Gainesville, FL 32610.; 3 Department of Biostatistics, College of Medicine; University of Florida, Gainesville, FL 32610.; 4 Department of Medicine, College of Medicine; University of Florida, Gainesville, FL 32610.

**Keywords:** pancreas, digital spatial profiling, tumor microenvironment, stromal interaction, immune tolerance

## Abstract

**Rationale:** The biology of the pancreatic ductal adenocarcinoma (PDAC) is heterogenous, but how heterogenity of the tumor microenvironment contributes to disparate patient outcomes remains essentially unstudied.

**Methods:** A strategy employing multiplex digital spatial profiling (mplxDSP) technology was employed to evaluate the nature and dynamics of microenvironment components including cancer associated fibroblasts (CAFs) and infiltrating immune cells at the single-cell level based upon their spatial relationship within the tumor.

**Results:** We report that myofibroblasts directly adjacent to PDAC tumors comparatively overexpress genes (BATF3, IL12B, ITGB8, CD4 and IFNAR1), constructing pathways prone to stimulating an adaptive immune response. Markers of innate immune cells (Natural Killer cells, Dendritic Cells and macrophages) are predominant in CD45+ cells immediately adjacent to PDAC tumor, however, the checkpoint protein CTLA4 is also overwhelmingly expressed, fostering tolerance. Finaly, mRNA profiling of adjacent CAFs identified clusters of genes that correlate with survival.

**Conclusion:** CAFs and leukocytes in close proximity to PDAC significantly differ from those remote from the tumor, providing insight into microenvironment influence on immune tolerance mediated through relative populations of leukocytes and subsets of CAFs and monocytes. mRNA expression profiling of CAFs adjacent to PDAC cells may hold promise for prognostication.

## Introduction

There is a fascinating, relatively unique feature of pancreatic ductal adenocarcinoma (PDAC): its mass is usually comprised of less than 20% epithelial tumor cells, and is mostly comprised of surrounding stroma. This desmoplastic stromal reaction in PDAC is majorly attributed to the activation of quiescent stellate cells becoming alpha-smooth muscle actin (α-SMA) expressing myofibroblasts, often termed as cancer-associated fibroblasts (CAFs) or tumor-associated stromal (TAS) cells. Initial studies of the impact of α-SMA^+^ cells suggested they contributed to disease progression by promoting cell proliferation and invasion as well as drug resistance 1, 2. However, this concept has recently been challenged as depletion of α-SMA^+^ myofibroblasts in genetically-engineered murine models of pancreas cancer accelerates tumor progression 3; Genetic reduction or pharmacologic inhibition of sonic hedgehog (Shh) pathway resulted in reduced stromal content with more aggressive tumors in KPC mice 4, 5, and a similar phenomenon was simultaneously reported in type I collagen (Col1) deficient tumors 6. We previously reported TAS cells express and deliver tumor suppressive miRNA-145 to neighboring PDAC cells 7, offering a potential mechanism for these observations. More recent studies identified distinct stromal types and subtypes of α-SMA^+^ cells suggesting that stromal heterogenicity associates with tumor development and progression 8-10. For example, Hutton, et al. reported CD105^pos^ pancreatic fibroblasts are typically more abundant in PDAC tumors and tumor permissive, whereas CD105^neg^ fibroblasts suppress tumor growth in a manner dependent on adaptive immunity 11. The heterogeneity of pancreatic fibroblast has raised attention in recent studies. Several research groups have reported different signature markers that could define pancreatic fibroblasts functions.

Yet, the nature and dynamics of α-SMA^+^ cells and their controversial role in PDAC tumor microenvironment remain poorly elucidated.

This fibrotic PDAC microenvironment is also highly immunosuppressive, conducive for tumor development and growth. In general, the PDAC microenvironment is equipped with upregulated tumor supporting cells [i.e., myeloid-derived suppressor cells (MDSCs), tumor-associated macrophages (TAMs), regulatory T cells (Tregs), etc.], while tumor destroying cells are downregulated [i.e., nature killer (NK) cells, CD8+ cells, etc.] 12. Recent work from Carstens, *et al.* utilized a novel computational imaging technology for spatially-characterized, heterogeneous T lymphocytes subpopulations infiltration in PDAC. The study delivered an important message of utilizing spatial information to improve our understanding of the heterogenous tumor microenvironment with respect to its correlation with patient outcome, specifically for stratifying PDAC patients for effective immunotherapy 13.

In this study, we aimed to investigate how spatial relationships between the three major components of PDAC including PDAC cells, activated myofibroblasts (CAFs), and immune cells contributes to heterogeneity within those compartments using novel multiplex digital spatial profiling (mplxDSP) technology. We further hypothesized that spatial-driven analyses of these contributors to the microenvironment may offer prognostic value.

## Methods

### Patients

Four Patients of PDAC who underwent surgical resection at the University of Florida (UF) Department of Surgery were enrolled in this study following UF Institutional Review Board (IRB) approved protocols. All patients were white race with no smoking history. Pathological diagnosis was confirmed to be invasive, moderately differentiated PDAC. Main clinical features of these patients are summarized in Supplemental Table 1. Metastasis was absent in all patients at time of resection. None received neoadjuvant chemotherapy or radiation. Patients were selected and grouped based on the clinical outcomes: reported disease associated death within approx. 12 months after surgical resection due to recurrence (Rcr) and patients with prolonged overall survival longer than 36 months after surgical resection with no reported recurrence, grouped as no disease evidence (NDE).

### Morphologic markers labeling and regions of interest (ROIs selection)

**Figure 1:**
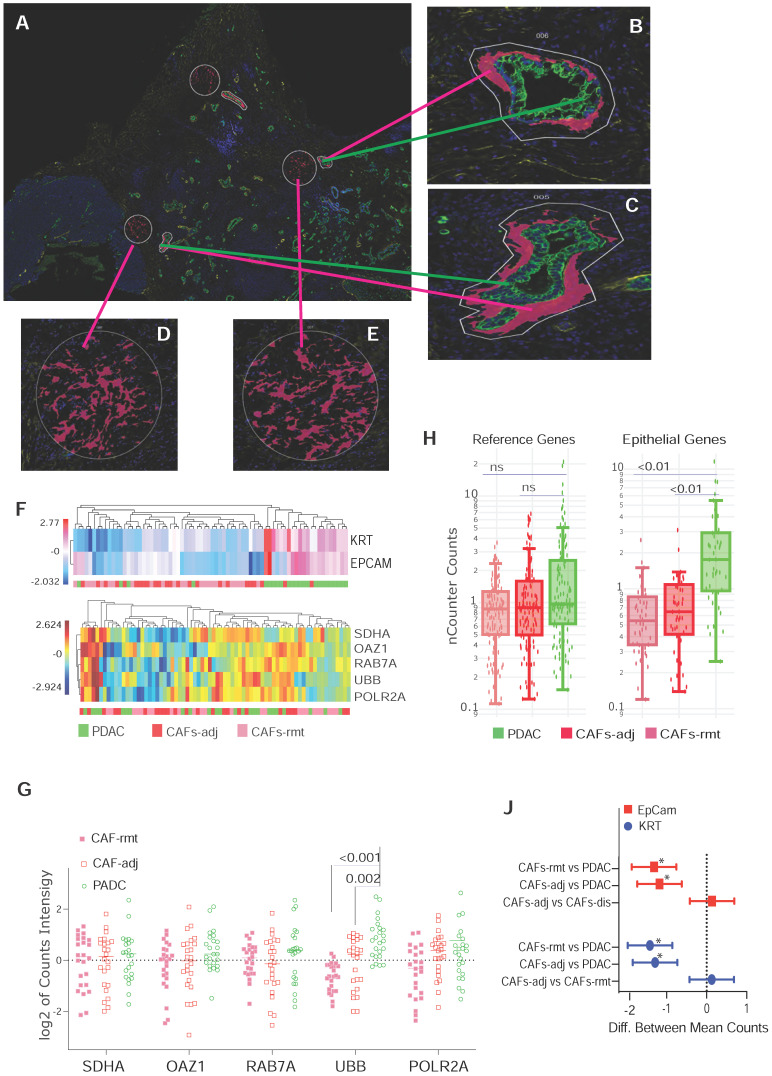
** ROIs selection and characterization for CAFs by locolization. (A-E)** Representative ROIs selection using immunofluorescent staining for morphological markers. **(B & C)** Enlarged images of PDAC cells with panCK antibody labeling, green-colored and immediate adjacented CAFs (CAF-adj) labeled with α-SMA antibody, red-colored. **(D & E)** CAFs distant to tumor lesion (CAF-rmt) also labeled with α-SMA antibody, red-colored. **(F)** Dendrogram of unsupervised hierarchically clustered analysis of the expression of epithelial genes of Epcam and KRT in log2 scale (top panel) and the expression of reference genes SDHA, OAZ1, RAB7A, UBB and POLR2A (bottom panel). **(G)** Dot plot of the expression level of the 5 reference genes individually by two way ANOVA, y-axil indicates log2 scale of reading intensity; displayed numbers are p-value. **(H)** Box plot of non-parametric analysis of total nCount counts for the set of four reference genes (SDHA, OAZ1, RAB7A, and POLR2A) and the two epithelial genes (EpCan and panCK); displayed numbers are p-value, ns = not significant. **(J)** Line plot of 95% Confidence Intervals (CI) of 2-way ANOVA multiple comparison of between PDAC cells, CAF-adj and CAF-rmt for EpCam and KRT; * = p < 0.001.

Formalin-fixed and paraffin-embedded (FFPE) tumor blocks were preserved at the time of surgical resection. One of the 5-µm serial sections from each tissue was stained for histological analysis of hematoxylin and eosin (HE) and used as reference for ROIs selection. DSP morphologic labeling for ROIs selection for three single cell ROIs using fluorescently labeled antibodies specific for total immune cells (anti-CD45), epithelial cell marker (anti-panCK), and (anti-αSMA), along with SYTO 13 DNA dye. A total of 24 ROIs for each cell types were defined and selected, except for tumor protein profiling, which used 12 ROIs. Examples are shown in Figures 1A-E and 3A-F. All ROIs were identified within serial sections from the same tumor core. Quality control was performed within the GeoMx DSP analysis.

### mplxDSP transcripts and protein panel

To quantitate gene expression at mRNA level, a minimum of 300 µm surface area of one ROI were selected. The nanoString GeoMx^®^ Immune Pathways RNA panel consists of 78 genes, including 73 target genes and 5 internal reference genes. This gene panel covers a number of cytokines and chemokine signaling molecules, key genes involved in tumorigenesis and tumor immunology (The complete gene list and annotations is listed in Suppl. Table S2, provided by nanoString, Sept 2019 MK1810). For measuring protein expression, we used the nanoString GeoMx^®^ Immune Cell Profiling Panel, which consists of a panel of 21 proteins including 3 housekeeping proteins (Suppl. Table S3). High-resolution multiplex profiling for selected cells followed the manufacturer's instructions. Photocleaved probes were hybridized to nanoString optical barcodes for digital counting and quantified in nCounter platform. Final digital counts were normalized with internal spike-in controls to account for system variation and background was corrected using the negative probe controls.

### Enhanced pathway Analysis

The differentially expressed genes identified between groups were input into the online software of Ingenuity Pathway Analysis (IPCA; www.integenuity.com) to curate gene interactions and correlation networks. A core analysis was employed to identify the most significant canonical pathways, molecular functions and network interactions. Pathways with p value <0.05 was considered statistcally significant.

### Statistical Analysis

To identify differential expressed genes, we used the linear model followed by the empirical Bayes moderated t-statistics test (two tailed), which are implemented in the R software *limma* package 14. We employed the Benjamini-Hochberg correction 15 to adjust for the multiple comparison and the false discovery rate (q-value) was reported. Unsupervised clustering analysis was performed using hierarchical clustering analysis. Dimension reduction was performed using principal component analysis. All statistical analyses were carried out using R (v. 4.1.1) software.

## Results

### RNA mplxDSP of spatially distributed αSMA+ CAFs in PDAC microenvironment

**Figure 2:**
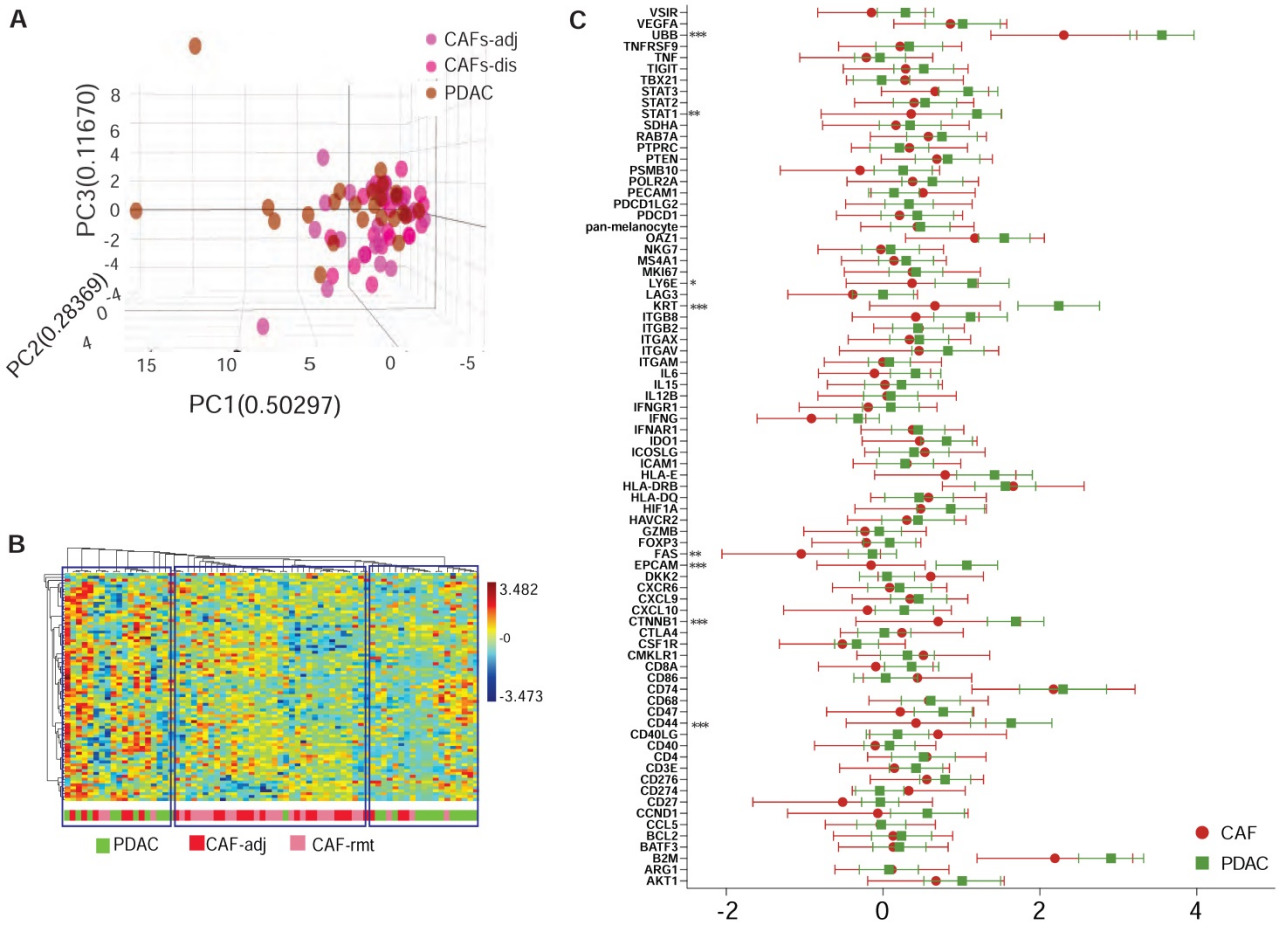
** Multiplex DSP for RNA profiling CAFs vs PDAC cells. (A)** Comparison profiling of 78 RNA gene expression of CAF and PDAC cells, * = p < 0.05, ** = p < 0.01, *** = p < 0.001. **(B)** Tri-dimentional diagam showing the result of principal component analysis (PCA) using the panel of 78 genes, each dot represent one ROI. **(C)** Dendrogram showing the unsupervised hierarchically cluster analysis of relative gene expression (y-axus) across all ROIs (x-axis).

We previously observed that CAFs immediately adjacent to transformed epithelial cells expressed more intensive α-SMA immunostaining 16. This observation was confirmed by others and contributed to the discovery of sub-populations of CAFs in the PDAC microenvironment by co-culture of murine PSCs and PDAC organoids 9. Our goal here was to test the capacity of spatially resolved gene profiling for defining heterogeneously distributed CAFs using mplxDSP technology. ROIs of CAF-rmt is chosen to avoid immediately next to PDAC tumor, the mean distance of a cluster of CAFs-rmt to the nearest PDAC epithelial compartment is at approximal of 331.1 µm (SD = 66.03). We profiled and compared 78 immuno-oncogenes from CAFs located immediately adjacent to PDAC epithelial compartment (adjacent CAFs, CAFs-adj) and CAFs located remote from cancer cells (PDAC distal CAFs, CAFs-rmt). Twenty-four individual regions of interest (ROIs) of each CAFs-adj and CAFs-rmt (defined by morphology marker anti-α-SMA antibody labeling) along with 24 ROIs of PDAC cells (defined by morphology marker anti-panCK antibody labeling) were selected for profiling (Figure 1A-E). The 78 RNA multiplex panel consists of 73 target genes and 5 reference genes (Suppl. Table S2). Unsupervised clustering analysis demonstrated the correspondent expression of epithelial genes of EpCam and KRT separating panCK+ PDAC cells from CAFs, despite few PDAC ROIs also expressing low level of epithelial expression (Figure 1F, top panel). As expected, no obvious cluster was assembled by the expression of the panel of 5 reference genes among all samples (Figure 1F, bottom panel). No differential expression of any of these genes was noticed between the two locations of CAFs. However, among the five reference genes (SDHA, OAZ1, RAB7A, UBB and POLR2A, Figure 1G), UBB alone was found differentially expressed with significant higher level in PDAC cells compared to CAFs (mean differences between CAFs-adj and CAFs-rmt to PDAC are -0.957 (p = 0.002) and -1.477 (p < 0.001) respectively). These data are in line with reported UBB upregulation in cancers 17, 18. We thus excluded UBB from the reference gene panel and the initial data were then normalized with the panel of the remaining four reference genes (of SDHA, OAZ1, RAB7A, and POLR2A) for further analysis. Based on the adjusted normalization, we observed no differential expression levels of total reference genes but distinguishable epithelial gene expressions between PDAC cells to CAFs-adj and CAFs-rmt (Figure 1H-J). Within the 73 target genes and 5 reference genes, 8 genes (approximal 10%) were detected with significantly higher expression levels in PDAC cells than CAFs (Suppl. Table S4 and figure 2C). Despite that this panel of 78 genes was not able to distinguish the two types of cells (Figure 2B), the hierarchical cluster analysis showed three dominate clusters: PDAC, CAFs and a mixture of both.

### Protein mplxDSP of spatially distributed CD45+ immune cells in PDAC microenvironment

**Figure 3:**
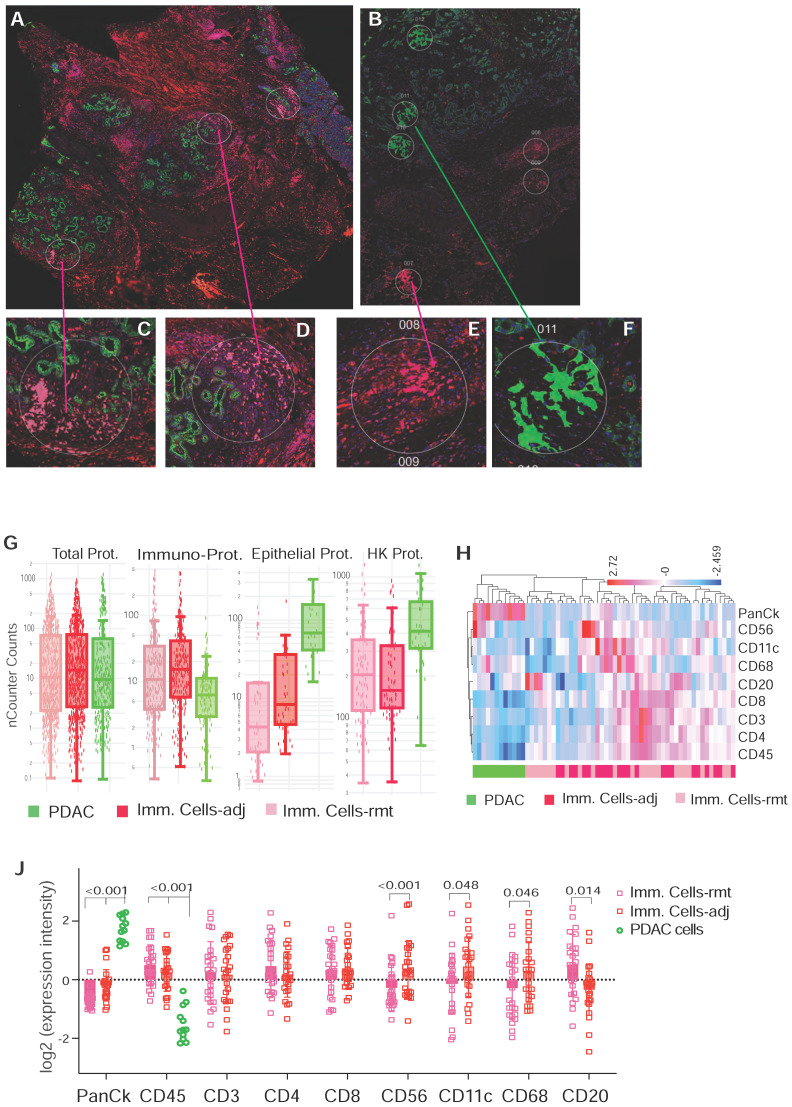
** Representative ROIs of CD45+ immune cells selection in PDAC microenvironment. (A & B)** Immunofluorescent staining of morphologic markers on PDAC tumor tissue. **(C, D & F)** Regions of interest (ROIs) of anti-panCK antibody labeled PDAC cells (green-colored) and immune cells in vicinity (Imm.Cells-adj, red-colored). **(E)** Regions of interest (ROIs) for selection of immune cells in remote area (Imm. Cells-rmt, red-colored). **(G)** Box plot of total nCounter Counts of multiplex DSP analysis for the total 18-plex proteins, CD45, panCK, and the three housekeeping proteins (panels from left to right). **(H)** Dendrogram showing distinct clusters of PDAC cells from immune cells -adj and -rmt based on the expression of markers; and **(J)** Expression levels of marker proteins determined by DSP from three sorts of ROIs for PDAC cells and immune cells adjacent and remote, displayed numbers are p-value.

**Figure 4:**
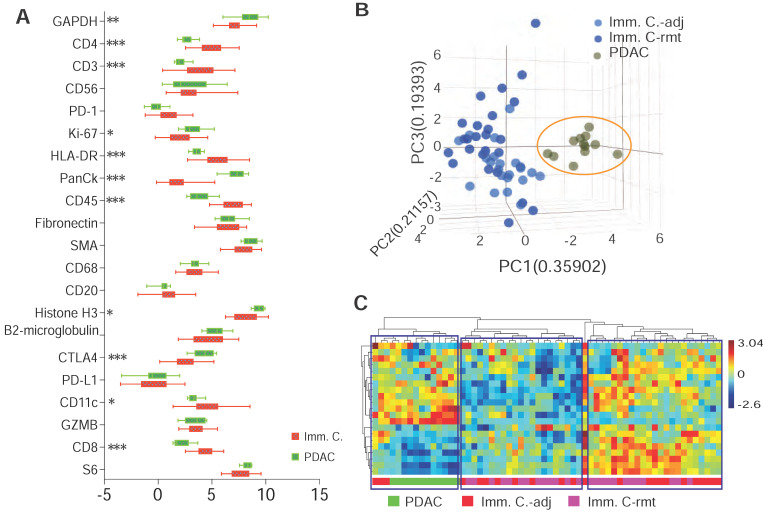
** DSP defines differential protein expression of PDAC cells from immune cells. (A)** Comparison profiling of multiplex DSP panel of 21 protein expression in selected CD45+ immune cells and panCK+ PDAC cells, * = p < 0.05, ** = p < 0.01, *** = p < 0.001. **(B)** A tri-dimention plot of PCA showing PDAC cells cluster from immune cells; and **(C)** Dendrogram of heiracherical cluster analysis. Three tantative clusters are boxed. Color bar indicates z-score value.

A computational method using immunohistochemistry spatial probing information showed that human PDAC contains a heterogeneous T-cell population 13. We employed mplxDSP technology to further characterize immune cells in PDAC microenvironment. The mplxDSP immune-protein panel (Suppl. Table S3) includes markers for total immune cells (CD45), subtypes of T-cells (CD3, CD4, CD8), Dendric cells (CD11c), B cells (CD20), Natural Killer cells (CD56) and M2 macrophage cells (CD68). We again divided immune cells into two groups by their localization to tumor lesion: CD45+ immune cells in immediate vicinity of PDAC area (immune cells-adjacent, Imm. Cells-adj) and those with no direct contact with PDAC cells (immune cells-remote, Imm. Cells-rmt). For each subtype of immune cells, 24 ROIs were selected and profiled to determine the relative abundance of these populations of immune cells. PDAC cells as counterpart compartment were also tested from 12 selected ROIs (Figure 3A-F & Suppl. Figure S2). As expected, total nCount counts (expression level) of markers for immune cells are found higher in CD45+ immune cells, both in Imm. Cells-adj and Imm. Cells-rmt in comparison to PDAC cells, and vice versa, the total nCount counts (expression level) of the epithelial pan-CK are high in PDAC cells and low in all CD45+ immune cells (Figure 3G). However, it is noticed that total nCount counts for the three housekeeping proteins (Histone H3, S6 and GAPDH) were also significantly more intensive in PDAC cells in comparison to immune cells (Figure 3G). Given that the total nCount counts of all proteins are even in both PDAC and immune cells, normalization was then performed based on the total protein counts. Demonstrated in Figure 3H, ROI selection successfully clustered two distinct segmentations of PDAC cells from immune cells, yet not between Imm.Cells-adj and Imm. Cells-rmt. As we further compared the subtypes of immune cells by distance to tumor lesion, we also observed the increased protein levels of NK cell marker CD56 (p < 0.001) and decreased B cell marker CD20 (p = 0.014) in immune cells adjacent (Imm.Cells-adj) to PDAC compared to those in distant stroma (Imm. Cells-rmt). DC marker CD11c (p = 0.048) and M2 macrophage marker CD68 (p = 0.046) were also seen slightly increased in Imm.Cells-adj. No differential protein expression of T cell markers, neither CD3, CD4, nor CD8, were observed (Figure 3J). Among the 3 housekeeping proteins and 18 target proteins, over 60% of proteins (11/18) from the panel were differentially expressed between PDAC and immune cells (Figure 4A). A group of PDAC cells was thus clearly separated from immune cells as indicated by PCA (Figure 4B) and hierarchical cluster analysis (Figure 4C). Thus, comparison of the Imm. Cells-adj to Imm. Cells-rmt demonstrated these quantities of these proteins are similar between the two ROIs, despite that some cells appears having more intensive expression than the others (Figure 4C).

### Leukopoiesis is enriched in CAFs adjacent to PDAC

**Figure 5:**
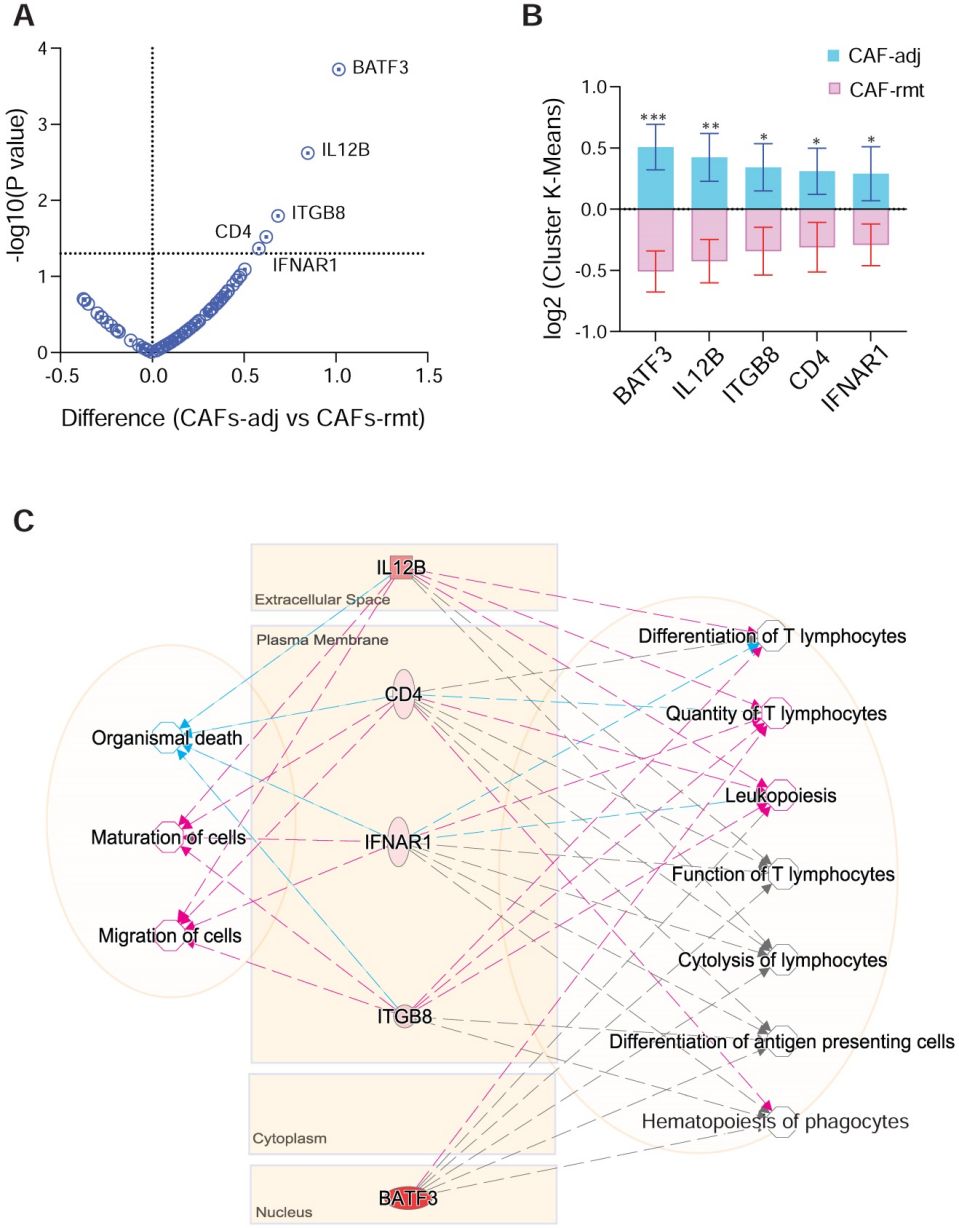
** DSP defines differential gene expression and pathway activities of CAFs by localisation. (A)** Volcano plot and **(B)** bar graft of statistical significance against cluster differential genes between CAFs-adj and CAFs-rmt; * p < 0.05, ** p < 0.01, *** p < 0.001; and **(C)** Gene-to-function relationship depicted by IPA. Color of lines and arrows correspondent to the relationship of genes to the annotated functions: red colored lines and arrows indicate increased function, blue colored lines and arrows indicate decreased function, and gray colored lines and arrows indicated that the gene is involved without further details.

We next compared target genes involved in tumor-immune pathways using mpxDSP mRNA 73-multiplex panel between CAFs in PDAC-adjacent (CAFs-adj) and remote (CAFs-rmt) locations. The key molecules in this panel are involved in tumor-immune biological activities, such as cytokine and chemokine signaling, Wnt signaling, tumor proliferation and apoptosis, especially in immune pathways of antigen presentation and checkpoint, myeloid suppression and activation, T cell activation, as well as immune cell adhesion and migration. Five genes (BATF3, IL12b, ITGB8, CD4 and IFNAR1) were found to have increased expression levels in CAFs-adj comparing to CAFs-rmt (Figure 5A-B). Interestingly, genes of IL12b, ITGB8, CD4 and IFNAR1 all have reportedly similar function to decrease Organismal Death (p value = 3.88E-03) 19-21, but increase Maturation of Cells (p value = 2.94E-06) 22-24, and Migration of Cells (p value = 2.37E-03) 25. The set of genes are also known affecting immune cells differentiation and function, highlighted as increased Quantify of T lymphocytes (p value = 6.42E-08) 26-29, Differentiation of T lymphocytes (p value = 1.61E-06), and Leukopoiesis (p value = 5.01E-07) 30-33. Despite known involvement in Hematopoiesis of Phagocytes (p value = 1.42E-07) 32, Function of T lymphocytes (p value = 1.71E-07) 34, Differentiation of Antigen Presenting cells (p value = 1.78E-07) 35 and Cytolysis of lymphocytes (p value = 1.31E-09) 36, their precise role in these functions is yet not clear (Figure 5C).

### Checkpoint protein CTLA4 overexpression in immune cells adjacent to PDAC

**Figure 6:**
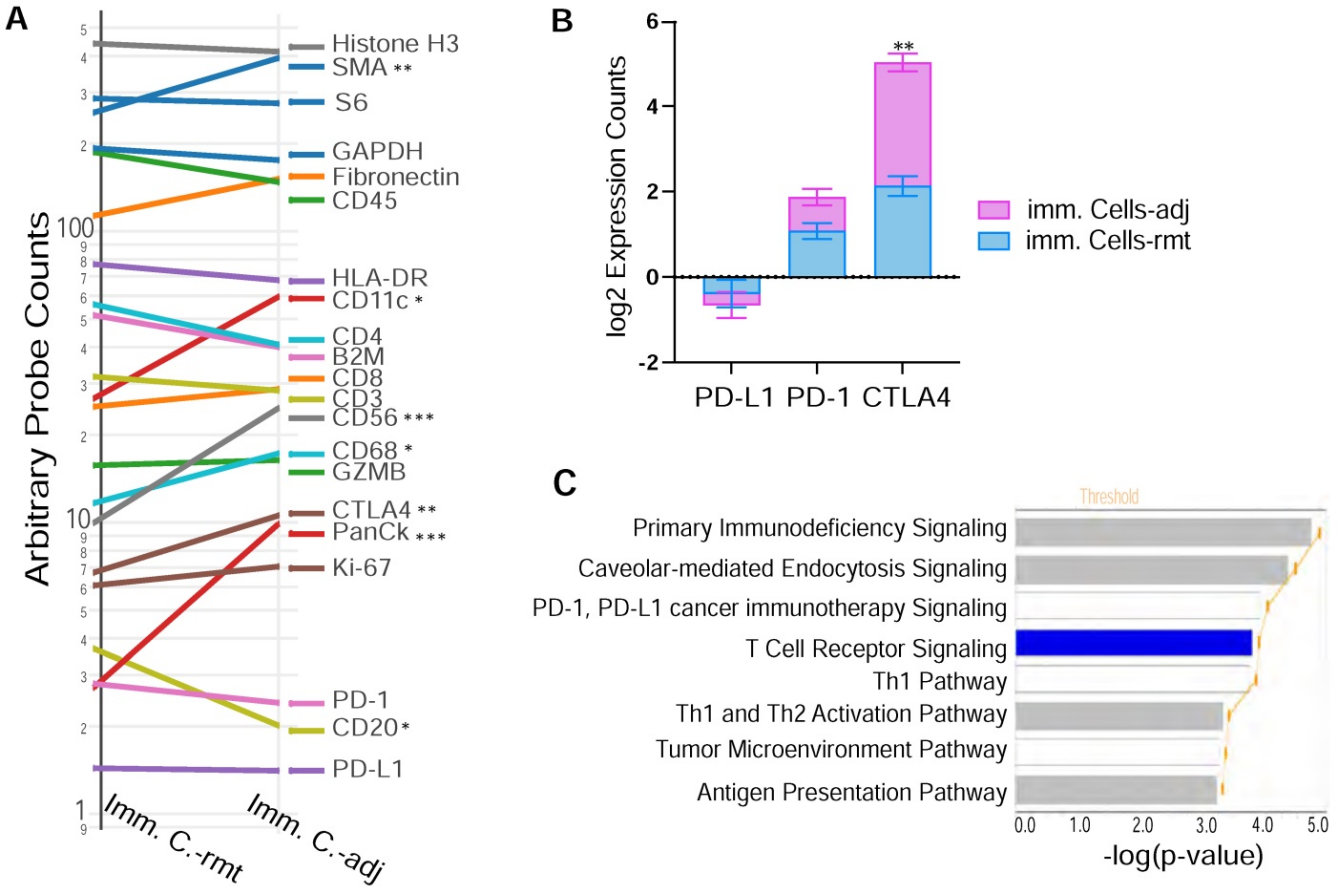
** DSP defines differential CTLA4 protein expression in PDAC adjacent immune cells. (A)** Trend line plot to compare the eighteen target genes and three housekeeping genes expression between immunce cells adjacent (imm. C.-adj) and remote (imm. C.-rmt) to PDAC area. X-axis is grouped segments of ROIs and y-axis plots for nCount probe counts. * p < 0.05, ** p < 0.01, *** p < 0.001; **(B)** bar graft of the protein expression for the three checkpoint proteins PD-L1, PD-1 and CTLA4 in both immunce cells -adj and -rmt; ** p < 0.01. **(C)** The top significant function annotations predicted by IPA show impoverished T Cell Receptor Signalling pathway in immune cells adjacent to PDAC. The significance of canonical pathways was determined with default threshold of -log(p-value) = 1.3 (p-value = 0.05).

Figure 6A demonstrates the expression of 18 target proteins plus 3 housekeeping proteins in the nanoString™ immuno-oncology proteins panel from both immune cells adjacent and remote to PDAC cells. As shown by the trend comparison, the three housekeeping proteins (Histone H3, GAPDH and S6) together with the 2 stromal proteins (SMA and Fibronectin), as well as total immune cell marker CD45 are strongly expressed in both locations of immune cells adjacent (imm. C.-adj) and remote (imm. C.-rmt), with only SMA highly expressed in PDAC adjacent immune cells (p < 0.01). Level of pan-CK (a cocktail of anti-KRT1, KRT10, KRT2, KRT16, KRT5, KRT6B, KRT19, KRT6A, KRT8, KRT14, KRT3 antibodies) was also significantly higher in immune cells adjacent to PDAC (p < 0.001). As described above in Figure 6A, NK cell marker CD56 is expressed at higher levels in PDAC adjacent immune cells by trend analysis, but B cell marker protein CD20 is found expressed at lower levels in PDAC adjacent immune cells. Noticeably, checkpoint PD-L1 and PD-1 have relatively lower expression levels and are not differentially expressed between PDAC adjacent (imm. C.-adj) and remote (imm. C.-rmt) immune cells. However, checkpoint protein CTLA4 expression was found expressed at moderate levels and considerably higher in PDAC adjacent immune cells (imm. C.-adj) compared to remote immune cells (Figure 6A & 6B), which is also a major contributor to downregulated T cell receptor signaling pathway predicted by IPA (p = 1.29E-04).

### Multiplex DSP with potential for recurrence prediction

**Figure 7:**
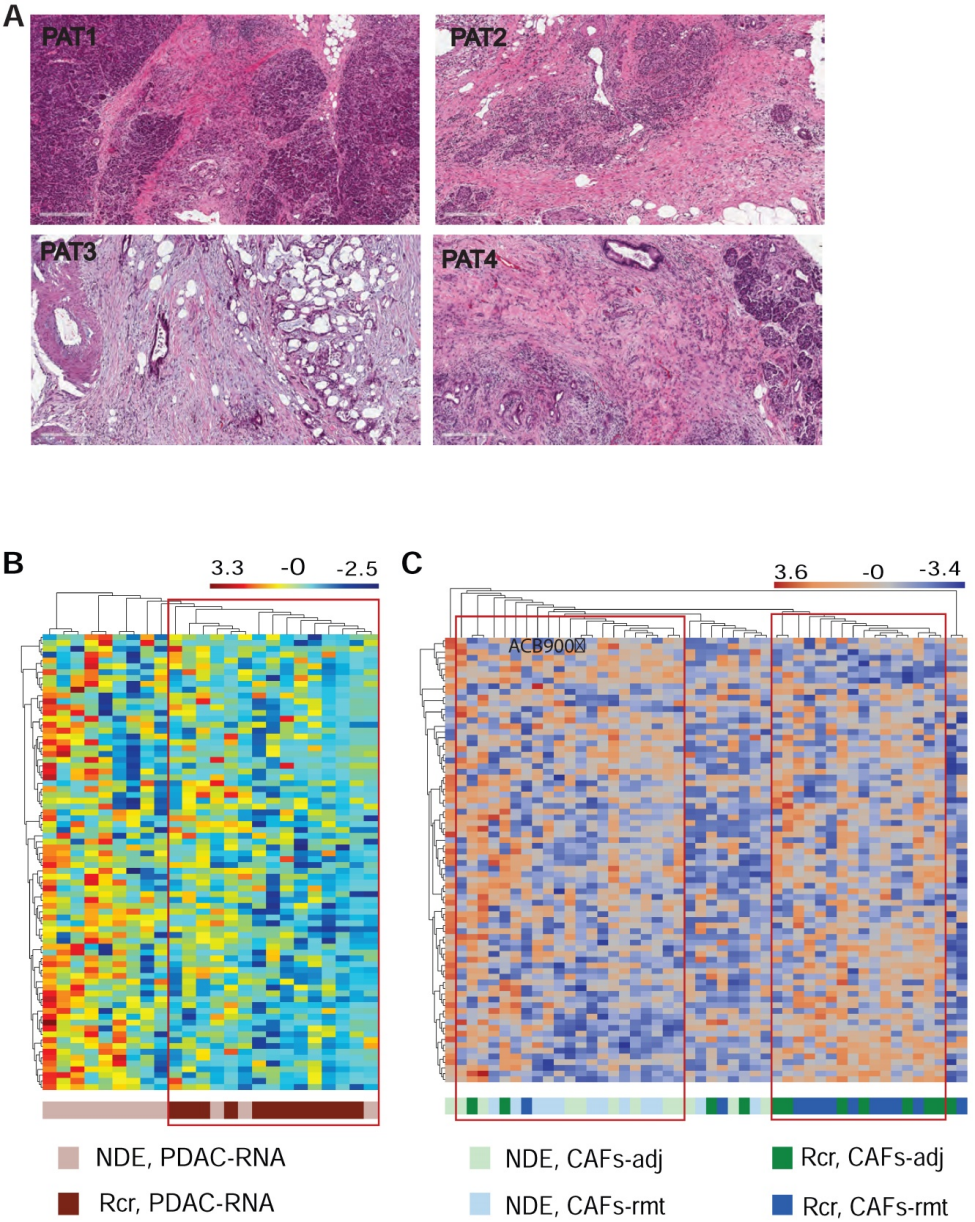
** Differential gene expression and pathway activation. (A)** HE staining of FFPE of the four resected tumors; **(B & C)** heatmap cluster dendrograms of RNA profiling panCK+ PDAC cells and for αSMA+ CAFs respectively.

Lastly, we explored whether mplexDSP, with the capability of single cell profiling, can surpass the bulk tissue profiling and provide better insights in predicting recurrence and patient survival. Four patients who underwent pancreaticoduodenectomy (Whipple surgery) were recruited in the study. Pathologic examination confirmed diagnosis of invasive, moderately or moderately to poorly differentiated PDAC (Suppl. Table S1 & Figure 7A). Among them, two patients encountered recurrence and death within 14 months (Rcr), and the other two patients experienced longer survival (over 36 months) with no reported recurrence (termed as no disease evidence, NDE) at the time of last follow up. Using the panel of 81-RNA data from mplexDSP, we observed tentative clusters that potentially distinguish patients with recurrence from patients with no reported recurrence. As suggested by Figure 7B, PDAC cell RNA multiplex showed a clearer cluster consisting all ROIs (12/12, 100%) from recurrent patients with mixture of 20% (3/15) ROIs from patients without recurrence. Profiling this gene set on SMA+ stromal cells also demonstrated tentative separation of recurrence from no disease evidence. As shown in Figure 7C, three tentative clusters are identified: first a Rcr-ROIs formed cluster including 66.7% of ROIs from recurrent tumors (16/24,); a second NDE-ROIs-dominated cluster including 87.5% (18/21) of ROIs from tumors of no disease evidence, with 18 of NDE-ROIs (18/24, 66.7%) and 3 Rcr-ROIs (3/24, 12.5%); lastly, a small cluster of 8 ROIs mingled across both with and without recurrence patient samples. Despite the lack of tightness of perfect clusters, both single cell analysis of PDAC or stromal cells displayed better heatmap construction in comparison to mixed-cell profiling (Suppl. Figure S1).

## Discussion

Here we performed a study that simultaneously determines gene and protein expression from single cells of the three major cell types within the pancreatic tumor microenvironment using FFPE tumor sections. Despite the small cohort of patients, our data reveal insightful and informative cues of the tumor-stroma-immune contexture, highlighting the potential power of such approach as a novel tool to better understand PDAC tumor biology and for biomarker discovery.

We, and others, have observed the phenomenon of PDAC tumors defined by stromal cells with varying intensity of αSMA expression depending upon their proximity to the epithelial cancer cells 9, 16. Using an organoid and PSC co-culture model, Öhlund et al. defined two distinct subtypes of CAFs based on their distance to tumor: the αSMA^high^ IL-6^low^ periglandular myofibroblasts (myCAFs) and more distantly distributed αSMA^low^ IL-6^high^ inflammatory CAFs (iCAFs) 9. More importantly, the authors reported the two subtypes are reversible, indicating a dynamic feature of CAFs. In this work, using the mplxDSP technology, we were able to perform spatial transcriptional profiles on human tissue using FFPE sections. Accordingly, differentially expressed transcripts are found between adjacent or remote αSMA+ myofibroblasts, supporting the theory that the PDAC microenvironment is comprised of distinct phenotypes of stromal cells. The pathway analysis of our data suggests that the periglandular stromal cells likely participate in recruiting immune cells and stimulating immune cell differentiation and expansion. Despite the reported conflicting function of αSMA+ myofibroblasts, there is increased awareness that stroma demonstrate an interplay of tumor promoting and tumor suppressing effects 37. It is now rational to believe that this phenomenon could be attributed to spatial distribution of CAFs. This hypothesis is well supported by the recent discovery of subTMEs from the study of Grünwald et al., the existence of “reactive” and “deserted” subTME associated with fibroblast plasticity 38.

In addition to αSMA+ myofibroblasts, we also analyzed the microenvironment immune cell distributions and their associated properties. Representative work by Carstens, et al. used computational, 8-color IHC multispectral imaging to discover that the distribution of the total numbers of T cell and subpopulation of T cells differs by location in relation to tumor, and more importantly, correlates with PDAC patient survival 13. Our approach of mplxDPS measures the levels of infiltrated immune cells by the transcript expression intensity, including both T cells and innate immune cells. While we observed no significant spatial distribution of T cell subpopulations, we detected an increase in the innate immune compartments, e.g. macrophage, DC and NK cells, in immune cells directly adjacent to PDAC tumor. In the setting of cancer, innate immunity has been documented to both promote and inhibit T cell priming and effector activity 39. Alas, we are not in a position to test the function of these innate immune cells in this work, however, our data raise support further investigation of how the proximity of innate immune components relative to the tumor may be important to T cells in influencing and shaping the known immunosuppressive PDAC microenvironment.

Therapeutic targeting of immune checkpoint molecules through inhibition of CTLA-4, PD-1 and PD-L1 has led to a paradigm shift in the treatment of many solid tumors 40, but has shown less encouraging outcomes in the treatment of PDAC 41. There is increased awareness of the impact of the expression profile of immune checkpoints on immune-modulatory therapies to specific individuals. Although a consensus view that successful treatment depends on optimal patient selection, reaching this goal remains a challenge without a guiding biomarker. For example, Steele et al. comprehensively characterized the tumor microenvironment and found that CD8^+^ T cells expressed markedly immune checkpoint profiles in individual patient and did not cluster by disease stage 42. Our interesting finding of CTLA-4 overexpression (but not PD1 or PDL1) in immune cells adjacent to PDAC tumor cells, provides compelling evidence that spatial information might be used to guide immunotherapy selection, but this requires additional investigation.

There are still many questions left to answer: The function of αSMA+ myofibroblasts beyond a simple physical barrier as conventionally acknowledged; The involvement of all tumoral and stromal components that contribute to the immunosuppressive PDCA microenvironment; The strategy of spatially defined single cell biomarkers for improving disease prognostication or the application of precision medicine, etc. We demonstrate here an initial effort toward uncovering these answers and also demonstrated the great advantages of mplxDSP technology.

## Conclusion

Our work has provided a novel insight into the distinguishable phenotypes of non-tumor cells that inscribe the heterogenicity of the pancreatic tumor microenvironment. How to utilize spatial biomarkers for clinical benefit awaits further exploration and validation with larger patient cohorts.

## Supplementary Material

Supplementary figures.

Supplementary tables.
