# Supplementary material for: Single-cell profiling of microenvironment components by spatial localization in pancreatic ductal adenocarcinoma
Source: Theranostics. 2022 Jun 27;12(11):4980–92. doi: 10.7150/thno.73222 (PMC9274743; doi:10.7150/thno.73222)
Supplement: Supplementary file 1 — Supplementary figures. [file thnov12p4980s1.pdf]

A

B

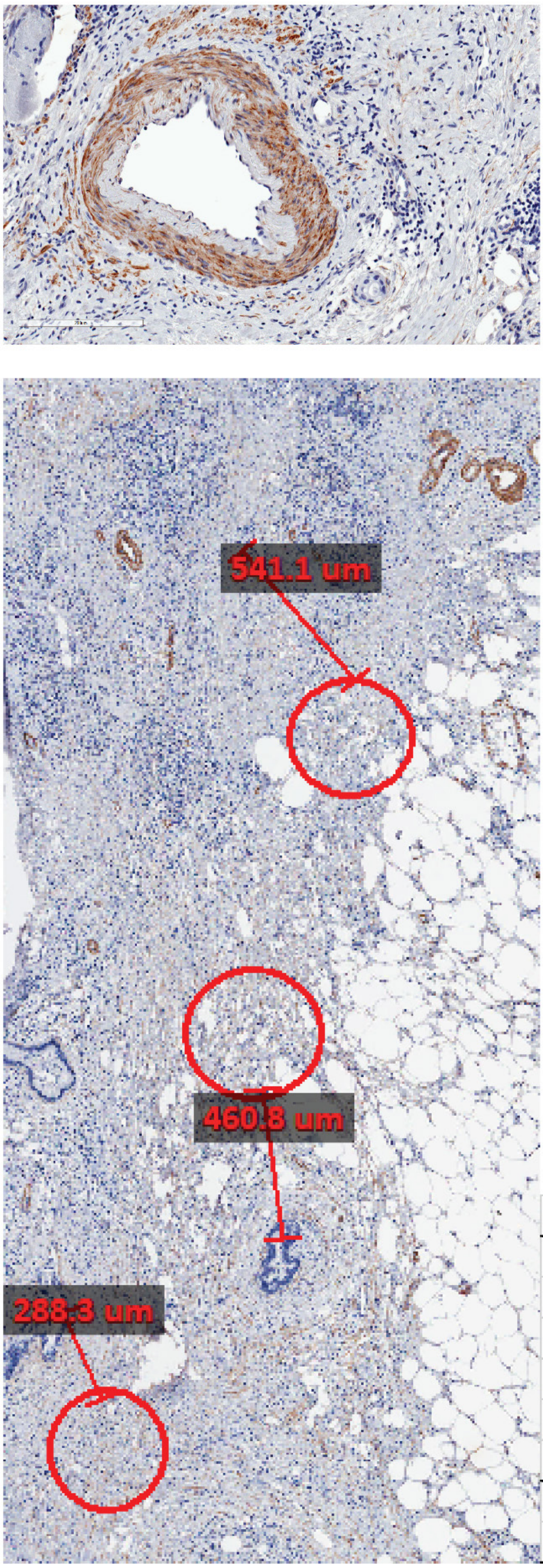

	PAT1	PAT2	PAT3	PAT4	total
ROI-1	325.3	271.7	313.3	541.1	
ROI-2	442.3	283.8	140.6	460.8	
ROI-3	369.3	288.1	432.5	288.3	
ROI-4	503.6	227.7	177.3	269.5	
ROI-5	207.8	342.8	351.4	404.4	
ROI-6	407	210.8	302.8	466.8	
Average (um)	375.883	270.817	286.317	405.15	334.542
SD	102.513	47.1396	109.292	107.178	66.0333

Figure S1: Selection of ROIs for CAF-adj and CAF-rmt. Immunohistochemistry (IHC) staining for α-SMA on FFPE slides shows the intensive expression on CAF-adj (A) and remote CAFs were carefully selected to ensure no nearby PDAC tumors involved, and distance (μm) between CAF-rmt to the nearest PDAC tumors were measured (B).

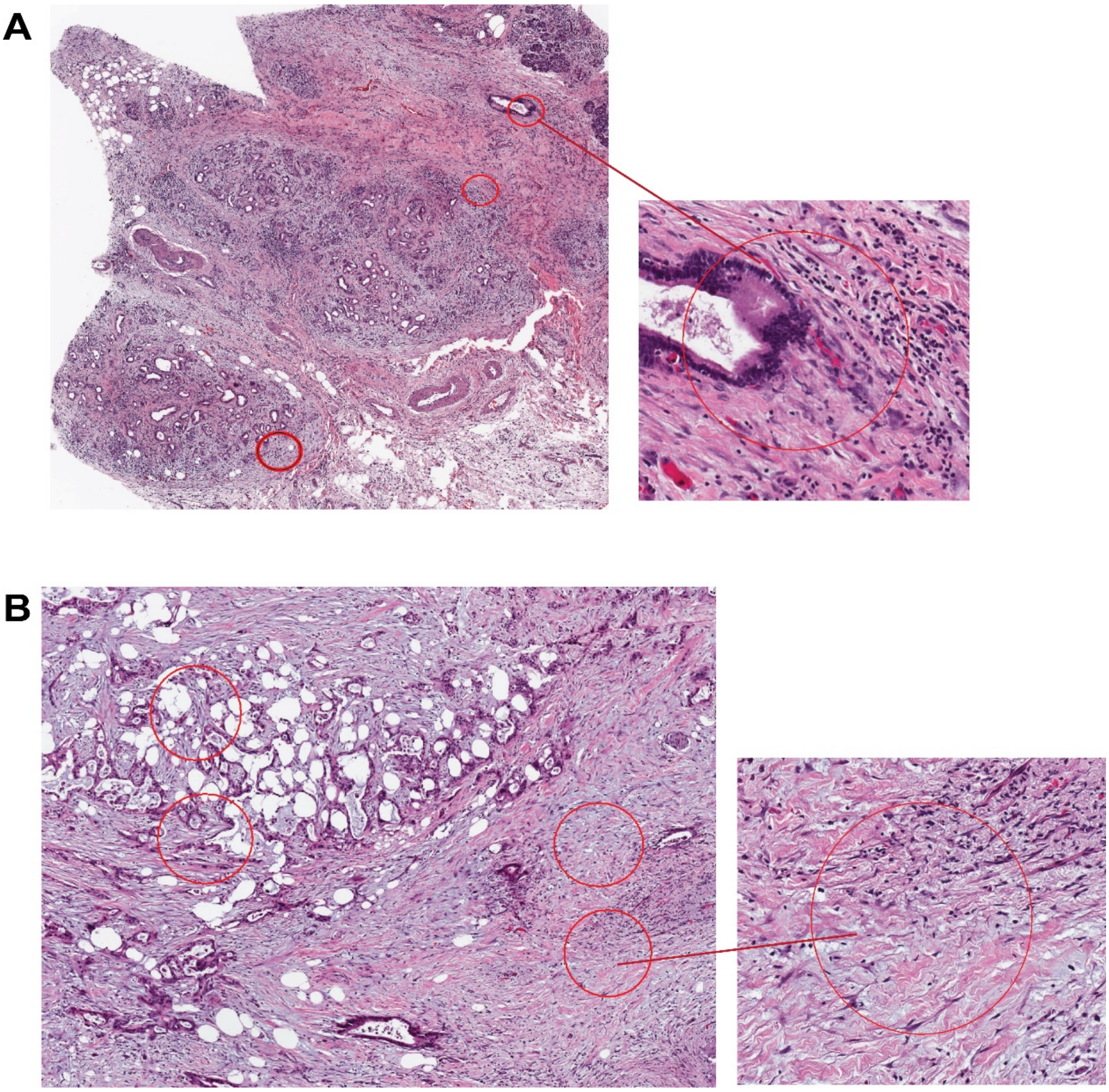

Figure S2: Correspondent hematoxylin and eosin (H&E) staining for ROIs selection for adjacent and remote immune cells. Representative H&E staining of chosen ROIs for Imm. Cells-adj (A) and Imm. Cell-rmt (B). Magnification for whole tissue images is 4x and 20x for enlarged images.

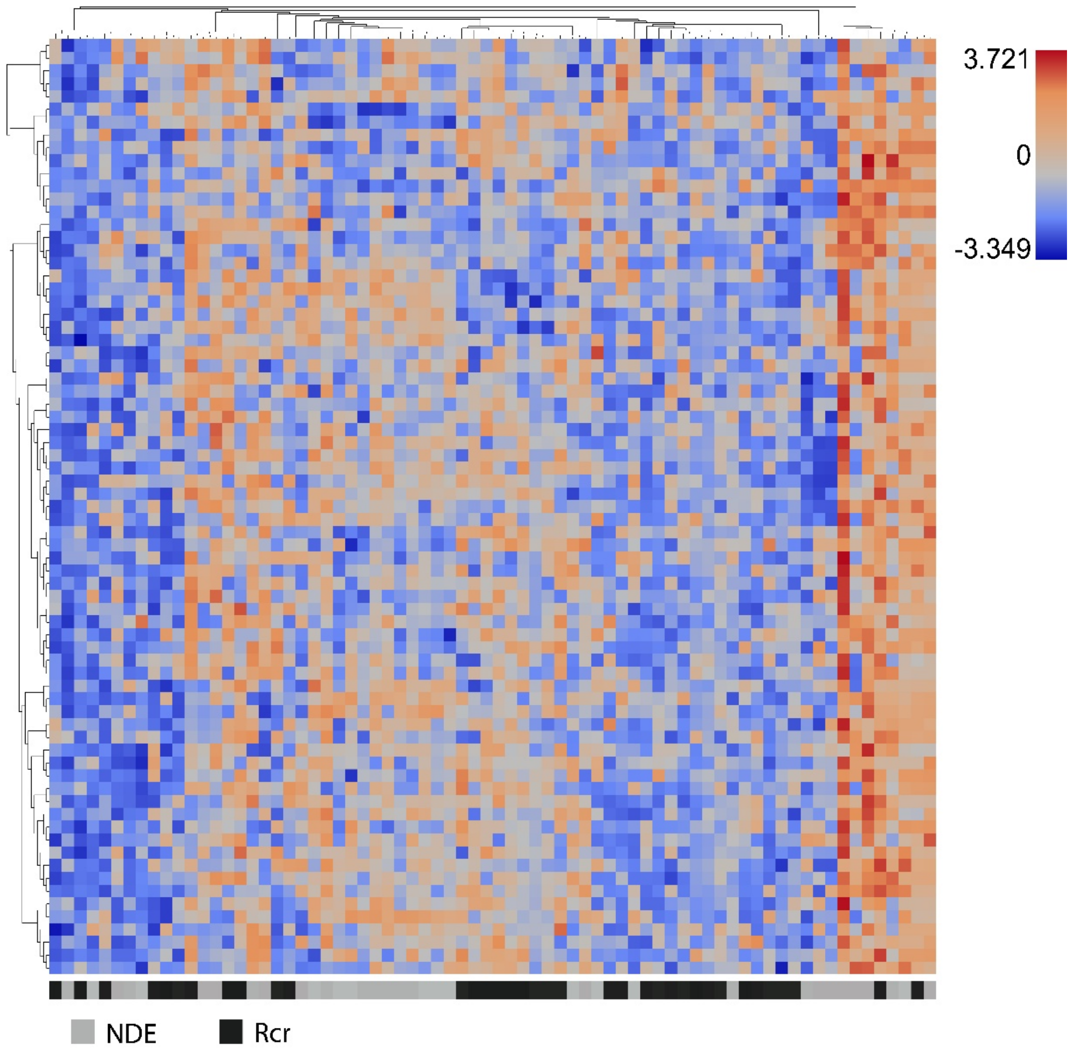

Figure S3: Correlation of recurrence with Bulk cells gene profiling. Heatmap of hierarchical clustering analysis based on the set of 81-RNA expression from mixture of the two types of cells of PDAC tumor cells and CAFs (column) versus 72 ROIs of recurrence (black symbols) and NDE (no disease evidence, gray symbols).
