# Supplementary material for: Single-cell profiling of microenvironment components by spatial localization in pancreatic ductal adenocarcinoma
Source: Theranostics. 2022 Jun 27;12(11):4980–92. doi: 10.7150/thno.73222 (PMC9274743; doi:10.7150/thno.73222)
Supplement: Supplementary file 2 — Supplementary tables. [file thnov12p4980s2.pdf]

**Suppl. Table 1 | Patients' Information**

| Group ID | Early Recurrence (Rcr) | | No Disease Evidence (NDE) | |
|---|---|---|---|---|
| **Patient ID** | **PAT1** | **PAT2** | **PAT3** | **PAT4** |
| Age | 65 | 64 | 80 | 78 |
| Gender | M | F | M | F |
| Race | W | W | W | W |
| Diabetes | No | No | No | No |
| Pre-op EUS/FNA | Yes | Yes | Yes | Yes |
| Biliary stenting | Yes | Yes | Yes | Yes |
| T-stage | 3 | 3 | 3 | 3 |
| N-stage | 1 | 1 | 1 | 0 |
| Differentiation | Moderately - poorly | moderately | moderately | moderately |
| Positive nodes (#) | 9 | 2 | 2 | 0 |
| T-Size (cm) | 3.2 | 3.8 | 3.8 | 2.5 |
| Pathology | PDAC | PDAC | PDAC | PDAC |
| Cause of death | Recurrence | Recurrence | N/A | N/A |
| Survival-Death (days) | 409 | 169 | 1124 | 1146 |

## Suppl. Table 2 - GeoMx® Immune Pathways RNA Profiling Panel

| Probe Name | Gene(s) | Accession(s) | Alias(es) | Full Name |
|---|---|---|---|---|
| **Target Genes** | | | | |
| ARG1 | ARG1 | NM_000045.3,NM_001244438.2,NR_160934.1,NM_001369020.1,NM_000045.4 | arginase, liver | arginase 1 |
| B2M | B2M | NM_004048.2,XM_005254549.3,XR_002957658.1,NM_004048.3 | | beta-2-microglobulin |
| BATF3 | BATF3 | NM_018664.2,XR_001737289.1,XR_921869.2,XM_017001683.1,NM_018664.3 | basic leucine zipper transcription factor, ATF-like 3 | basic leucine zipper ATF-like transcription factor 3 |
| BCL2 | BCL2 | NM_000633.2,NM_000657.2,XR_935248.3,XM_011526135.3,XM_017025917.2 | B-cell CLL/lymphoma 2,BCL2, apoptosis regulator | BCL2 apoptosis regulator |
| CCL5 | CCL5 | NM_002985.2,NM_002985.3,NM_001278736.2 | D17S136E,SCYA5,small inducible cytokine A5 (RANTES),chemokine (C-C motif) ligand 5 | C-C motif chemokine ligand 5 |
| CCND1 | CCND1 | NM_053056.2 | BCL1,D11S287E,PRAD1,cyclin D1 (PRAD1: parathyroid adenomatosis 1) | cyclin D1 |
| CD27 | CD27 | NM_001242.4,XM_017020232.1,XM_017020234.1,XM_011521042.3,XM_017020233.2 | TNFRSF7,tumor necrosis factor receptor superfamily, member 7 | CD27 molecule |
| CD274 | CD274 | NM_014143.3,NR_052005.1,NM_014143.4,NM_001314029.2,NM_001267706.1 | PDCD1LG1,programmed cell death 1 ligand 1,CD274 antigen | CD274 molecule |
| CD276 | CD276 | NM_001024736.1,XM_017022638.1,XM_005254700.4,XM_011522095.2,NM_001024736.2,NM_001329629.2,NM_025240.2,NM_001329628.2 | CD276 antigen | CD276 molecule |
| CD3E | CD3E | NM_000733.3 | CD3e antigen, epsilon polypeptide (TiT3 complex),CD3e molecule, epsilon (CD3-TCR complex) | CD3e molecule |
| CD4 | CD4 | NM_000616.4,XM_017020228.2,NM_000616.5,NM_001195016.3,NM_001195017.3,NM_001195014.3,NM_001195015.3 | CD4 antigen (p55),T-cell surface glycoprotein CD4 | CD4 molecule |
| CD40 | CD40 | NM_001250.5,NR_126502.1,NR_136327.1,XM_017028136.1,XM_011529109.2,XM_005260619.3,XM_017028135.1,NM_001322422.2,NM_001322421.2,NM_152854.4,NM_001250.6,NM_001302753.2,NM_001362758.2 | TNFRSF5,tumor necrosis factor receptor superfamily, member 5,CD40 molecule, TNF receptor superfamily member 5 | CD40 molecule |
| CD40LG | CD40LG | NM_000074.2 | HIGM1,IMD3,TNFSF5,tumor necrosis factor (ligand) superfamily, member 5 (hyper-IgM syndrome) | CD40 ligand |
| CD44 | CD44 | NM_001001392.1,XM_005253238.3,XM_017018585.2,XM_011520485.2,XM_011520484.2,XM_005253240.3,XM_011520486.2,XM_011520488.2,XM_005253232.3,XM_011520483.2,XM_017018583.2,XM_006718390.4,XM_005253235.3,XM_006718388.2,XM_017018584.2,XM_011520487.3,XM_011520489.3,XM_011520482.2,XM_005253231.3,XM_005253239.3,NM_001202557.2,NM_001001389.2,NM_001202556.2,NM_001001390.2,NM_001202555.2,NM_000610.4,NM_001001391.2,NM_001001392.2 | MIC4,MDU2,MDU3,CD44 antigen (homing function and Indian blood group system) | CD44 molecule (Indian blood group) |
| CD47 | CD47 | NM_198793.2,NM_001777.3,XR_241521.2,XR_241522.2,XM_005247909.2,XR_002959610.1,XR_924218.2,XR_001740374.2,XR_924219.2,XR_924220.2,XR_002959611.1,XR_001740375.2,XM_005247908.2 | MER6,CD47 antigen (Rh-related antigen, integrin-associated signal transducer) | CD47 molecule |
| CD68 | CD68 | NM_001251.2,NM_001251.3,NM_001040059.2 | CD68 antigen | CD68 molecule |
| CD74 | CD74 | NM_001025159.2,NM_004355.3,NM_001025158.2,XR_002956198.1,XM_017010089.2,XM_017010090.2,NR_157074.1,NM_001364083.2,NM_001364084.2 | DHLAG,CD74 antigen (invariant polypeptide of major histocompatibility complex, class II antigen-associated),CD74 molecule, major histocompatibility complex, class II invariant chain | CD74 molecule |
| CD86 | CD86 | NM_001206924.1,NM_001206925.1,NM_006889.4,NM_175862.5,NM_176892.1 | CD28LG2,CD86 antigen (CD28 antigen ligand 2, B7-2 antigen) | CD86 molecule |
| CD8A | CD8A | NM_001145873.1,NM_001768.6,NM_171827.3,NR_027353.1 | CD8,CD8 antigen, alpha polypeptide (p32),T-cell surface glycoprotein CD8 alpha chain | CD8a molecule |
| CMKLR1 | CMKLR1 | NM_001142345.1,NM_004072.2,XM_017018820.2,NM_001142343.2,NM_001142344.2,NM_004072.3 | chemokine-like receptor 1 | chemerin chemokine-like receptor 1 |
| CSF1R | CSF1R | NM_005211.3,NR_109969.1,NM_001288705.2,NM_001349736.1 | FMS,McDonough feline sarcoma viral (v-fms) oncogene homolog | colony stimulating factor 1 receptor |
| CTLA4 | CTLA4 | NM_001037631.2,NM_001037631.3,NM_005214.5 | CELIAC3,IDDM12,celiac disease 3,insulin-dependent diabetes mellitus 12 | cytotoxic T-lymphocyte associated protein 4 |
| CTNNB1 | CTNNB1 | NM_001098209.1,XM_006712985.1,XM_017005738.1,XM_024453356.1,XM_006712983.2,XM_024453360.1,XM_024453358.1,XM_024453357.1,XM_024453359.1,NM_001904.4,NM_001330729.2,NM_001098210.2,NM_001098209.2 | CTNNB,catenin (cadherin-associated protein), beta 1 (88kD),catenin (cadherin-associated protein), beta 1, 88kDa,catenin (cadherin-associated protein), beta 1 | catenin beta 1 |
| CXCL10 | CXCL10 | NM_001565.3,NM_001565.4 | INP10,SCYB10,small inducible cytokine subfamily B (Cys-X-Cys), member 10,chemokine (C-X-C motif) ligand 10 | C-X-C motif chemokine ligand 10 |
| CXCL9 | CXCL9 | NM_002416.2,NM_002416.3 | CMK,MIG,monokine induced by gamma interferon,chemokine (C-X-C motif) ligand 9 | C-X-C motif chemokine ligand 9 |
| CXCR6 | CXCR6 | NM_006564.1,XM_005264809.2,XM_011533291.2,XM_011533290.2,NM_006564.2 | chemokine (C-X-C motif) receptor 6 | C-X-C motif chemokine receptor 6 |
| DKK2 | DKK2 | NM_014421.2,NM_014421.3 | dickkopf (Xenopus laevis) homolog 2,dickkopf 2 homolog (Xenopus laevis) | dickkopf WNT signaling pathway inhibitor 2 |
| EPCAM | EPCAM | NM_002354.2 | M4S1,MIC18,TACSTD1,antigen identified by monoclonal antibody AUA1,tumor-associated calcium signal transducer 1 | epithelial cell adhesion molecule |

| | | | | |
|---|---|---|---|---|
| FAS | FAS | NR_028033.3,NM_000043.5,NR_028035.3,NR_028036.3,NR_028034.3,XR_945733.2,XM_006717819.3,XM_011539765.2,XM_011539764.2,XM_011539766.2,XM_011539767.3,XR_945732.3,NM_000043.6,NM_152871.4,NR_135313.2,NM_001320619.2,NR_135315.2,NR_135314.2,NM_152872.4 | FAS1,APT1,TNFRSF6,tumor necrosis factor receptor superfamily, member 6,Fas (TNF receptor superfamily, member 6) | Fas cell surface death receptor |
| FOXP3 | FOXP3 | NM_014009.3,XM_006724533.2,XM_017029567.1,NM_001114377.2 | IPEX,immune dysregulation, polyendocrinopathy, enteropathy, X-linked | forkhead box P3 |
| GZMB | GZMB | NM_004131.5,NR_144343.1,NM_004131.6,NM_001346011.2 | CTLA1,CSPB,granzyme B (granzyme 2, cytotoxic T-lymphocyte-associated serine esterase 1) | granzyme B |
| HAVCR2 | HAVCR2 | NM_032782.4,NM_032782.5 | | hepatitis A virus cellular receptor 2 |
| HIF1A | HIF1A | NM_181054.2,NM_001243084.1,NM_001530.4,NM_181054.3 | hypoxia inducible factor 1, alpha subunit (basic helix-loop-helix transcription factor) | hypoxia inducible factor 1 subunit alpha |
| HLA-DQ | HLA-DQA1 | NM_002122.3,NM_002122.4,XM_006715079.4 | HLA-DQA | major histocompatibility complex, class II, DQ alpha 1 |
| HLA-DRB | HLA-DRB1,HLA-DRB3,HLA-DRB5,HLA-DRB4,HLA-DPB1 | NM_002124.3,NM_022555.3,NM_001359193.1,NM_001243965.1,XM_011514562.2,XM_024452554.1,XR_002958969.1,XR_002958970.1,XM_024452553.1,NM_002125.4,NM_021983.4,XM_011548129.2,XM_011548128.2,XR_952975.2,XM_011548127.2,NM_001359194.1,XM_024452550.1,XM_017030290.2,NM_002121.6 | HLA-DR1B,HLA-DR3B,HLA-DR4B | major histocompatibility complex, class II, DR beta 1,major histocompatibility complex, class II, DR beta 3,major histocompatibility complex, class II, DR beta 4 |
| HLA-E | HLA-E | NM_005516.5,XM_017010807.1,NM_005516.6,XM_017010808.1,XM_017010809.2 | | major histocompatibility complex, class I, E |
| ICAM1 | ICAM1 | NM_000201.2,NM_000201.3 | | intercellular adhesion molecule 1 |
| ICOSLG | ICOSLG,LOC102723996 | NM_015259.5,XM_006723899.2,XM_011546079.1,XM_011546078.2,XM_011529515.3,XM_024452060.1,XM_011529514.3,XM_011529516.3,NM_001283050.2,NM_001365759.2,NM_001363770.2,NM_015259.6,NM_001283052.2,NM_001283051.2 | ICOSL,inducible T-cell costimulator ligand | inducible T cell costimulator ligand |
| IDO1 | IDO1 | NM_002164.5,NM_002164.6 | IDO,INDO,indoleamine-pyrrole 2,3 dioxygenase | indoleamine 2,3-dioxygenase 1 |
| IFNAR1 | IFNAR1 | NM_000629.2,XM_005260964.2,NM_000629.3,XM_011529552.2 | IFNAR,interferon (alpha, beta and omega) receptor 1 | interferon alpha and beta receptor subunit 1 |
| IFNG | IFNG | NM_000619.2,NM_000619.3 | | interferon gamma |
| IFNGR1 | IFNGR1 | NM_000416.2,XM_011535794.1,XM_011535793.2,XM_006715470.3,NM_001363526.1,NM_001363527.1 | IFNGR | interferon gamma receptor 1 |
| IL12B | IL12B | NM_002187.2 | NKSF2,interleukin 12B (natural killer cell stimulatory factor 2, cytotoxic lymphocyte maturation factor 2, p40) | interleukin 12B |
| IL15 | IL15 | NM_000585.4,NR_037840.2,NM_000585.5,NM_172175.3 | | interleukin 15 |
| IL6 | IL6 | NM_000600.4,XM_011515390.2,XM_005249745.5,NM_000600.5,NM_001318095.2 | IFNB2,interleukin 6 (interferon, beta 2) | interleukin 6 |
| ITGAM | ITGAM | NM_000632.3,XM_006721045.1,XR_950796.1,XM_011545851.2,XM_017023216.1,XM_011545850.2,NM_000632.4,NM_001145808.2 | CR3A,CD11B,integrin, alpha M (complement component receptor 3, alpha; also known as CD11b (p170), macrophage antigen alpha polypeptide),integrin, alpha M (complement component 3 receptor 3 subunit) | integrin subunit alpha M |
| ITGAV | ITGAV | NM_001144999.2,NM_002210.5,NM_001145000.3 | VNRA,MSK8,VTNR,antigen identified by monoclonal antibody L230,vitronectin receptor,integrin, alpha V (vitronectin receptor, alpha polypeptide, antigen CD51),integrin, alpha V | integrin subunit alpha V |
| ITGAX | ITGAX | NM_000887.4,XM_011545852.1,XM_011545854.1,XR_950797.2,XM_024450263.1,NM_000887.5,NM_001286375.2 | CD11C,integrin, alpha X (antigen CD11C (p150), alpha polypeptide),integrin, alpha X (complement component 3 receptor 4 subunit) | integrin subunit alpha X |
| ITGB2 | ITGB2 | NM_000211.4,XM_006724001.2,NM_000211.5,NM_001303238.2,NM_001127491.3 | CD18,MFI7,integrin, beta 2 (antigen CD18 (p95), lymphocyte function-associated antigen 1; macrophage antigen 1 (mac-1) beta subunit),integrin, beta 2 (complement component 3 receptor 3 and 4 subunit) | integrin subunit beta 2 |
| ITGB8 | ITGB8 | NM_002214.2,XM_011515396.1,XM_017012181.1,XM_017012178.1,XM_011515394.2,XM_017012182.1,XM_011515393.2,XM_017012180.1,XM_017012179.1,XM_017012183.1,NM_002214.3 | integrin, beta 8 | integrin subunit beta 8 |
| KRT | KRT18,KRT10,KRT17,KRT19,KRT6A,KRT6B,KRT6C,KRT7,KRT14 | NM_000224.2,NM_199187.1,NM_000224.3,NM_000421.3,NM_000421.4,NM_000422.2,NM_000422.3,NM_002276.4,NM_002276.5,NM_005554.3,NM_005554.4,NM_005555.4,NM_173086.5,NM_005556.3,XM_017019294.1,XR_001748699.2,XR_001748700.2,XM_011538325.2,NM_005556.4,XM_005257343.3,NM_000526.4,NM_000526.5 | keratin 18, type I,KPP,keratosis palmaris et plantaris,keratin 10, type I,PCHC1,keratin 17, type I,keratin 19, type I,KRT6C,KRT6D,keratin 6C,keratin 6D,keratin 6A, type II,keratin 7, type II,EBS3,EBS4,keratin 14 (epidermolysis bullosa simplex, Dowling-Meara, Koebner),keratin 14, type I | keratin 18,keratin 10,keratin 17,keratin 19,keratin 6A,keratin 7,keratin 14 |
| LAG3 | LAG3 | NM_002286.5,XM_011520956.1,NM_002286.6 | lymphocyte-activation gene 3 | lymphocyte activating 3 |
| LY6E | LY6E | NM_001127213.1,NM_002346.3 | lymphocyte antigen 6 complex, locus E | lymphocyte antigen 6 family member E |
| MKI67 | MKI67 | NM_002417.4,XM_011539818.2,NM_002417.5,NM_001145966.2,XM_006717864.3 | antigen identified by monoclonal antibody Ki-67 | marker of proliferation Ki-67 |
| MS4A1 | MS4A1 | NM_021950.3,NM_152866.2 | CD20,membrane-spanning 4-domains, subfamily A, member 1 | membrane spanning 4-domains A1 |
| NKG7 | NKG7 | NM_005601.3,XM_006723228.3,XM_005258955.3,NM_005601.4,NM_001363693.2 | natural killer cell group 7 sequence | natural killer cell granule protein 7 |
| pan-melanocyte | PMEL,S100B,SOX10 | NM_001200054.1,NM_006928.4,NM_001200053.1,NM_001320122.1,NM_001320121.1,NM_006272.2,XM_017028424.2,NM_006272.3,NM_006941.3 | SIL,SILV,silver (mouse homolog) like,silver homolog (mouse),S100 calcium binding protein, beta (neural),SRY (sex determining region Y)-box 10,SRY-box 10 | premelanosome protein,S100 calcium binding protein B,SRY-box transcription factor 10 |

| | | | | |
|---|---|---|---|---|
| PDCD1 | PDCD1 | NM_005018.2,XM_017004293.1,NM_005018.3,XM_006712573.2 | SLEB2,systemic lupus erythematosus susceptibility 2 | programmed cell death 1 |
| PDCD1LG2 | PDCD1LG2 | NM_025239.3,XM_005251600.3,NM_025239.4 | | programmed cell death 1 ligand 2 |
| PECAM1 | PECAM1 | NM_000442.4,XM_005276880.1,XM_005276882.1,XM_005276881.1,XM_011524890.1,XM_017024741.1,XM_005276883.2,XM_017024738.1,XM_017024740.1,XM_017024739.1,XM_011524889.2,NM_000442.5 | platelet/endothelial cell adhesion molecule 1 | platelet and endothelial cell adhesion molecule 1 |
| PSMB10 | PSMB10 | NM_002801.3,NM_002801.4 | MECL1,proteasome (prosome, macropain) subunit, beta type, 10 | proteasome subunit beta 10 |
| PTEN | PTENP1,PTEN | NR_023917.1,NM_000314.6,NM_001304717.5,NM_000314.7,NM_001304718.2 | phosphatase and tensin homolog pseudogene 1 (functional) | phosphatase and tensin homolog pseudogene 1 |
| PTPRC | PTPRC | NM_080921.3,XM_006711474.3,XM_006711473.3,XM_006711472.4,NM_002838.5 | CD45 | protein tyrosine phosphatase receptor type C |
| STAT1 | STAT1 | NM_007315.3,NM_139266.2,XM_006712718.1,XR_001738915.2,XM_017004783.2,XR_001738914.2 | signal transducer and activator of transcription 1, 91kD,signal transducer and activator of transcription 1, 91kDa | signal transducer and activator of transcription 1 |
| STAT2 | STAT2 | NM_005419.3,XR_245953.3,XM_011538697.2,XM_011538700.2,XR_001748856.1,XR_001748857.1,XM_017019904.2,XR_002957375.1,XR_001748858.2,XM_011538699.3,XM_011538698.3,XR_002957376.1,NM_005419.4,NM_198332.2 | signal transducer and activator of transcription 2, 113kD,signal transducer and activator of transcription 2, 113kDa | signal transducer and activator of transcription 2 |
| STAT3 | STAT3 | NM_003150.3,NM_139276.2,XM_017024973.2,XM_024450896.1,NM_001369518.1,NM_003150.4,NM_001369519.1,NM_001369516.1,NM_001369517.1,NM_001369514.1,NM_001369512.1,NM_001369520.1,NM_001369513.1,NM_213662.2 | signal transducer and activator of transcription 3 (acute-phase response factor) | signal transducer and activator of transcription 3 |
| TBX21 | TBX21 | NM_013351.1,NM_013351.2 | T-box 21 | T-box transcription factor 21 |
| TIGIT | TIGIT | NM_173799.3,XR_002959502.1,XM_024453388.1,NM_173799.4 | VSIG9,VSTM3,VSIG9, VSTM3 | T cell immunoreceptor with Ig and ITIM domains |
| TNF | TNF | NM_000594.3,NM_000594.4 | TNFA,tumor necrosis factor (TNF superfamily, member 2) | tumor necrosis factor |
| TNFRSF9 | TNFRSF9 | NM_001561.5,XM_011541386.2,XM_006710618.3 | ILA,tumor necrosis factor receptor superfamily, member 9 | TNF receptor superfamily member 9 |
| VEGFA | VEGFA | NM_001171630.1,NM_001171625.1,NM_001171624.1,NM_001171626.1,NM_001171628.1,NM_001171629.1,NM_001171627.1,NM_001171623.1,NM_001204384.1,NM_001317010.1,NM_001025369.3,NM_001204385.2,NM_001025367.3,NM_001171622.2,NM_003376.6,NM_001025368.3,NM_001025370.3,NM_001033756.3,NM_001025366.3,NM_001287044.2 | VEGF,vascular endothelial growth factor | vascular endothelial growth factor A |
| VSIR | VSIR | NM_022153.1,NM_022153.2 | C10orf54,chromosome 10 open reading frame 54 | V-set immunoregulatory receptor |
| **Internal Reference Genes** | | | | |
| OAZ1 | OAZ1 | NM_004152.3,NM_001301020.1 | OAZ | ornithine decarboxylase antizyme 1 |
| POLR2A | POLR2A | NM_000937.4,NM_000937.5 | POLR2,polymerase (RNA) II (DNA directed) polypeptide A, 220kDa | RNA polymerase II subunit A |
| RAB7A | RAB7A | NM_004637.5 | RAB7,CMT2B,RAB7, member RAS oncogene family,Charcot-Marie-Tooth neuropathy 2B | RAB7A, member RAS oncogene family |
| SDHA | SDHA,SDHAP1,SDHAP2,LOC220729,SDHAP3 | NM_004168.2,NM_001294332.1,NM_001330758.1,XM_024446143.1,XR_002956167.1,XM_017009685.2,NM_004168.4,NM_004168.3,XM_011514073.2,XM_011514072.2,NR_003264.2,NR_003265.3,NR_003266.2,NR_003263.2 | SDH2,succinate dehydrogenase complex, subunit A, flavoprotein (Fp),succinate dehydrogenase complex subunit A, flavoprotein (Fp),SDHAL1,SDHALP1,succinate dehydrogenase complex, subunit A, flavoprotein-like 1,succinate dehydrogenase complex, subunit A, flavoprotein pseudogene 1,succinate dehydrogenase complex subunit A, flavoprotein pseudogene 1,SDHAL,SDHACL,succinate dehydrogenase complex, subunit A, flavoprotein-like,SDHA C-terminal like,succinate dehydrogenase complex, subunit A, flavoprotein pseudogene 3,succinate dehydrogenase complex subunit A, flavoprotein pseudogene 3 | succinate dehydrogenase complex flavoprotein subunit A,succinate dehydrogenase complex flavoprotein subunit A pseudogene 1,succinate dehydrogenase complex flavoprotein subunit A pseudogene 3 |
| UBB | UBB,UBBP4 | NM_001281716.1,NM_001281720.1,NM_018955.3,NM_001281717.1,NM_001281719.1,NM_001281718.1,NM_018955.4,NR_144546.1 | | ubiquitin B |

## Suppl. Table 3 - GeoMx® Immune Cell Protein Profiling Panel

| Protein Name | Accession | Full Target Name | Protein ID |
|---|---|---|---|
| **Target Proteins** | | | |
| CD68 | P34810 | CD68 molecule | DPROT_00006.1 |
| HLA-DR | P04233 | CD74 molecule, major histocompatibility complex, class II invariant chain | DPROT_00007.1 |
| Ki-67 | P46013 | marker of proliferation Ki-67 | DPROT_00009.1 |
| Beta-2-microglobulin | P61769 | beta-2-microglobulin | DPROT_00010.1 |
| CD11c | P20702,H3BN02 | integrin, alpha X (complement component 3 receptor 4 subunit) | DPROT_00011.1 |
| CD20 | A0A024R507,P11836 | membrane-spanning 4-domains, subfamily A, member 1 | DPROT_00012.1 |
| CD3 | B0YIY5,P09693,P07766,B0YIY4,P04234 | CD3g molecule, gamma (CD3-TCR complex) ,CD3e molecule, epsilon (CD3-TCR complex) ,CD3d molecule, delta (CD3-TCR complex) | DPROT_00013.1 |
| CD4 | B4DT49,P01730,B0AZV7 | CD4 molecule | DPROT_00014.1 |
| CD45 | M9MML4,A0A0A0MT22,P08575,X6R433 | protein tyrosine phosphatase, receptor type, C | DPROT_00015.1 |
| CD56 | P13591,A0A087WWD4 | neural cell adhesion molecule 1 | DPROT_00016.1 |
| CD8 | P01732,Q6ZVS2,Q8TAW8 | CD8a molecule | DPROT_00017.1 |
| CTLA4 | P16410 | cytotoxic T-lymphocyte associated protein 4 | DPROT_00395.1 |
| GZMB | J3KQ52,Q6XGZ4,P10144,Q67BC3 | granzyme B | DPROT_00019.1 |
| PD-L1 | Q0GN75,Q9NZQ7 | CD274 molecule | DPROT_00021.1 |
| PanCk | P04264,P13645,P35908,P08779,P13647,P04259,P08727,A0A0S2Z428,P02538,Q7L4M3,P05787,P02533,P12035 | keratin 1 ,keratin 10 ,keratin 2 ,keratin 16 ,keratin 5 ,keratin 6B ,keratin 19 ,keratin 6A ,keratin 8 ,keratin 14 ,keratin 3 | DPROT_00022.1 |
| SMA | D2JYH4,P62736 | actin, alpha 2, smooth muscle, aorta | DPROT_00023.1 |
| **Housekeepings** | | | |
| Fibronectin | B7ZLE5,P02751,Q6MZM7,Q9UQS6,Q6MZF4,Q6N084 | fibronectin 1 | DPROT_00024.1 |
| Histone H3 | P68431 | histone 1, H3a | DPROT_00005.1 |
| S6 | A2A3R6,P62753 | ribosomal protein S6 | DPROT_00008.1 |
| GAPDH | P04406,V9HVZ4 | glyceraldehyde-3-phosphate dehydrogenase | DPROT_00020.1 |

Suppl. Table S4 - DE (Differentially expressed) Genes between CAFs and PDAC cells

| Gene ID | Predicted (LS) mean diff. | 95.00% CI of diff. | Summary | Adj. P Value |
|---|---|---|---|---|
| AKT1 | -0.2889 | -1.006 to 0.4281 | ns | >.999 |
| ARG1 | 0.1241 | -0.5929 to 0.8411 | ns | >.999 |
| B2M | -0.754 | -1.471 to -0.03691 | * | 0.026 |
| BATF3 | -0.007809 | -0.7249 to 0.7092 | ns | >.999 |
| BCL2 | -0.1807 | -0.8977 to 0.5363 | ns | >.999 |
| CCL5 | -0.003357 | -0.7204 to 0.7137 | ns | >.999 |
| CCND1 | -0.6548 | -1.372 to 0.06224 | ns | 0.135 |
| CD27 | -0.5335 | -1.251 to 0.1836 | ns | 0.586 |
| CD274 | 0.3527 | -0.3643 to 1.070 | ns | >.999 |
| CD276 | -0.2009 | -0.9180 to 0.5161 | ns | >.999 |
| CD3E | -0.1762 | -0.8932 to 0.5408 | ns | >.999 |
| CD4 | 0.03733 | -0.6797 to 0.7544 | ns | >.999 |
| CD40 | -0.1788 | -0.8958 to 0.5383 | ns | >.999 |
| CD40LG | 0.439 | -0.2781 to 1.156 | ns | 0.947 |
| CD44 | -1.243 | -1.960 to -0.5262 | *** | <.001 |
| CD47 | -0.4952 | -1.212 to 0.2218 | ns | 0.769 |
| CD68 | -0.02783 | -0.7449 to 0.6892 | ns | >.999 |
| CD74 | -0.05368 | -0.7707 to 0.6634 | ns | >.999 |
| CD86 | 0.4292 | -0.2879 to 1.146 | ns | 0.963 |
| CD8A | -0.4655 | -1.183 to 0.2515 | ns | 0.881 |
| CMKLR1 | 0.2934 | -0.4236 to 1.010 | ns | >.999 |
| CSF1R | -0.1916 | -0.9086 to 0.5255 | ns | >.999 |
| CTLA4 | 0.3069 | -0.4102 to 1.024 | ns | >.999 |
| CTNNB1 | -0.9389 | -1.656 to -0.2218 | *** | <.001 |
| CXCL10 | -0.4363 | -1.153 to 0.2807 | ns | 0.952 |
| CXCL9 | -0.1486 | -0.8656 to 0.5685 | ns | >.999 |
| CXCR6 | -0.03438 | -0.7514 to 0.6827 | ns | >.999 |
| DKK2 | 0.6454 | -0.07161 to 1.362 | ns | 0.155 |
| EPCAM | -1.257 | -1.974 to -0.5396 | *** | <.001 |
| FAS | -0.8611 | -1.578 to -0.1441 | ** | 0.003 |
| FOXP3 | -0.2305 | -0.9475 to 0.4865 | ns | >.999 |
| GZMB | -0.1086 | -0.8256 to 0.6084 | ns | >.999 |
| HAVCR2 | -0.04067 | -0.7577 to 0.6764 | ns | >.999 |
| HIF1A | -0.3947 | -1.112 to 0.3224 | ns | 0.992 |
| HLA-DQ | 0.05858 | -0.6585 to 0.7756 | ns | >.999 |
| HLA-DRB | 0.1198 | -0.5972 to 0.8369 | ns | >.999 |
| HLA-E | -0.6245 | -1.342 to 0.09251 | ns | 0.209 |
| ICAM1 | 0.02983 | -0.6872 to 0.7469 | ns | >.999 |
| ICOSLG | 0.1168 | -0.6003 to 0.8338 | ns | >.999 |
| IDO1 | -0.2887 | -1.006 to 0.4284 | ns | >.999 |
| IFNAR1 | -0.1252 | -0.8423 to 0.5918 | ns | >.999 |

| | | | | |
|---|---|---|---|---|
| IFNG | -0.547 | -1.264 to 0.1701 | ns | 0.519 |
| IFNGR1 | -0.2027 | -0.9198 to 0.5143 | ns | >.999 |
| IL12B | 0.03619 | -0.6809 to 0.7532 | ns | >.999 |
| IL15 | -0.1079 | -0.8249 to 0.6092 | ns | >.999 |
| IL6 | -0.4944 | -1.211 to 0.2227 | ns | 0.772 |
| ITGAM | -0.05495 | -0.7720 to 0.6621 | ns | >.999 |
| ITGAV | -0.2409 | -0.9579 to 0.4762 | ns | >.999 |
| ITGAX | -0.1199 | -0.8369 to 0.5972 | ns | >.999 |
| ITGB2 | 0.000698 | -0.7163 to 0.7177 | ns | >.999 |
| ITGB8 | -0.6976 | -1.415 to 0.01942 | ns | 0.069 |
| KRT | -1.652 | -2.369 to -0.9346 | *** | <.001 |
| LAG3 | -0.4114 | -1.128 to 0.3057 | ns | 0.983 |
| LY6E | -0.6871 | -1.404 to 0.02991 | ns | 0.082 |
| MKI67 | 0.05844 | -0.6586 to 0.7755 | ns | >.999 |
| MS4A1 | -0.1234 | -0.8404 to 0.5937 | ns | >.999 |
| NKG7 | -0.2236 | -0.9406 to 0.4935 | ns | >.999 |
| OAZ1 | -0.3647 | -1.082 to 0.3523 | ns | 0.999 |
| pan-melanocyt | -0.07283 | -0.7899 to 0.6442 | ns | >.999 |
| PDCD1 | -0.1117 | -0.8288 to 0.6053 | ns | >.999 |
| PDCD1LG2 | 0.09882 | -0.6182 to 0.8159 | ns | >.999 |
| PECAM1 | 0.3174 | -0.3997 to 1.034 | ns | >.999 |
| POLR2A | -0.2558 | -0.9728 to 0.4613 | ns | >.999 |
| PSMB10 | -0.5415 | -1.259 to 0.1756 | ns | 0.546 |
| PTEN | -0.1109 | -0.8279 to 0.6062 | ns | >.999 |
| PTPRC | 0.1539 | -0.5631 to 0.8709 | ns | >.999 |
| RAB7A | -0.212 | -0.9291 to 0.5050 | ns | >.999 |
| SDHA | -0.08457 | -0.8016 to 0.6325 | ns | >.999 |
| STAT1 | -0.8192 | -1.536 to -0.1022 | ** | 0.008 |
| STAT2 | -0.1545 | -0.8716 to 0.5625 | ns | >.999 |
| STAT3 | -0.4829 | -1.200 to 0.2341 | ns | 0.82 |
| TBX21 | 0.3823 | -0.3348 to 1.099 | ns | 0.996 |
| TIGIT | -0.1245 | -0.8415 to 0.5926 | ns | >.999 |
| TNF | -0.1551 | -0.8722 to 0.5619 | ns | >.999 |
| TNFRSF9 | -0.1172 | -0.8342 to 0.5998 | ns | >.999 |
| UBB | -1.255 | -1.972 to -0.5375 | *** | <.001 |
| VEGFA | -0.1547 | -0.8718 to 0.5623 | ns | >.999 |
| VSIR | -0.4305 | -1.148 to 0.2865 | ns | 0.961 |
